# Parthenogenesis and Human Assisted Reproduction

**DOI:** 10.1155/2016/1970843

**Published:** 2015-11-09

**Authors:** Adriana Bos-Mikich, Fabiana F. Bressan, Rafael R. Ruggeri, Yeda Watanabe, Flávio V. Meirelles

**Affiliations:** ^1^Department of Morphological Sciences, ICBS, Federal University of Rio Grande do Sul, 90050-170 Porto Alegre, RS, Brazil; ^2^Department of Veterinary Medicine, Faculty of Animal, Science and Food Engineering (FZEA), University of São Paulo (USP), 13635-000 Pirassununga, SP, Brazil; ^3^WTA Fertilização In Vitro Ltda, 14140-000 Cravinhos, SP, Brazil

## Abstract

Parthenogenetic activation of human oocytes obtained from infertility treatments has gained new interest in recent years as an alternative approach to create embryos with no reproductive purpose for research in areas such as assisted reproduction technologies itself, somatic cell, and nuclear transfer experiments and for derivation of clinical grade pluripotent embryonic stem cells for regenerative medicine. Different activating methods have been tested on human and nonhuman oocytes, with varying degrees of success in terms of parthenote generation rates, embryo development stem cell derivation rates. Success in achieving a standardized artificial activation methodology for human oocytes and the subsequent potential therapeutic gain obtained from these embryos depends mainly on the availability of gametes donated from infertility treatments. This review will focus on the creation of parthenotes from clinically unusable oocytes for derivation and establishment of human parthenogenetic stem cell lines and their potential applications in regenerative medicine.

## 1. Introduction

Parthenogenesis is a reproduction strategy common in some jawed vertebrate species such as the whiptail lizard (*Aspidoscelis uniparens*) [1], in which no sperm is involved to trigger embryonic development from the oocyte and the female generates an offspring with no paternal inheritance. In mammals, parthenogenesis is not a natural form of reproduction, as the birth of an offspring is considered not possible. However, parthenogenetic activation of mammalian oocytes by artificial manipulation results in early embryonic development and in some instances fetal early development can be achieved (mouse forelimb stage E9.5) stage [2]. No further development is achieved due to the lack of expression of imprinted paternal genes necessary for the establishment of a functional placenta [3]. On the other hand, genetic modification technologies have made the creation and birth of fertile animals from mouse parthenogenetically activated oocytes possible [4].

Why then do we study parthenogenesis in the scope of assisted reproduction technologies (ART) if no human baby can be generated from this reproductive maneuver?

Renewed interest in parthenogenetic activation of mammalian oocytes has come about for scientific, medical, and economic reasons. First of all, experimental work involving parthenogenetic embryos circumvents the ethical and legal problems concerning the use of human embryos generated for reproductive purposes. Parthenotes may be employed in different research areas related to ART, studies on human pluripotent stem cells or basic science uncovering regulatory mechanisms that command human embryonic development and cloning experiments using somatic cell or nuclear transfer in mammalian oocytes.

Last but not least, the creation of clinical grade parthenogenetic human embryonic stem cell (hpESC) lines have the potential to benefit a considerable large number of patients, when used in cell therapies, with a reduced risk of transplant rejection. However, to this end, efforts should be directed to clinicians to participate in research projects. The establishment of parthenogenetic clinical grade human embryonic stem cell lines for use in cell or tissue therapies will only be possible, if research laboratories obtain enough biological material from ART centers to evaluate activation strategies, derivation, and culture media adequate for future therapeutic use of phESC lines. Furthermore, well trained committed staff, with good practice and knowledge of basic embryology, is paramount for the conscious and successful use of spare oocytes and embryos obtained from assisted reproduction centers.

This review will explore techniques of parthenogenetic activation, derivation, and cell and tissue differentiation achieved in animal models, which reinforce the therapeutic potential of these pluripotent embryonic stem cells, despite concerns related to their putative genetic instability, an inherent feature due to their monoparental origin [4].

**Figure 1:**
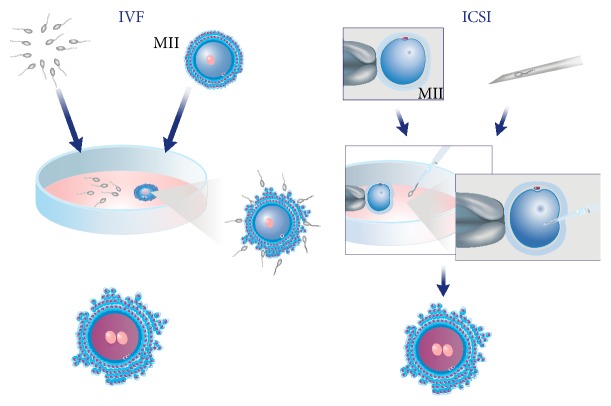
Oocyte insemination by* in vitro* fertilization (IVF) and intracytoplasmic sperm injection (ICSI) techniques.

*ART In Vitro Fertilization and Intracytoplasmic Sperm Injection Techniques*. Among the several treatments regarded as ART, there are two insemination methods employed for the generation of human embryos in the laboratory, the* in vitro* fertilization (IVF) and the intracytoplasmic sperm injection (ICSI) (Figure 1).

When performing IVF, oocytes and sperm are mixed together and left to undergo the fertilization process in a culture system that mimics the fallopian tubes environment. Thus, IVF may be considered an assisted reproduction technology that mimics “natural” fertilization phenomena [5]. With IVF, the fertilizing spermatozoon will penetrate through the surrounding* cumulus* cells, interact with the* zona pellucida* proteins, and eventually attach and fuse with the oocyte plasma membrane.

ICSI was developed to overcome situations where sperm count, motility, or morphology is compromised. By using this insemination technology, the embryologist may choose the “best looking” spermatozoon to inseminate the oocyte, even if it is in very low numbers or presents limited motility in the ejaculate.

ICSI is performed with the aid of a pair of glass pipettes adjusted to an inverted microscope, where the embryologist holds the oocyte with one pipette and injects the chosen spermatozoon with the second pipette straight into the ooplasm.

## 2. Parthenogenetic Activation Methodologies

### 2.1. Biochemistry of Oocyte Activation

**Figure 2:**
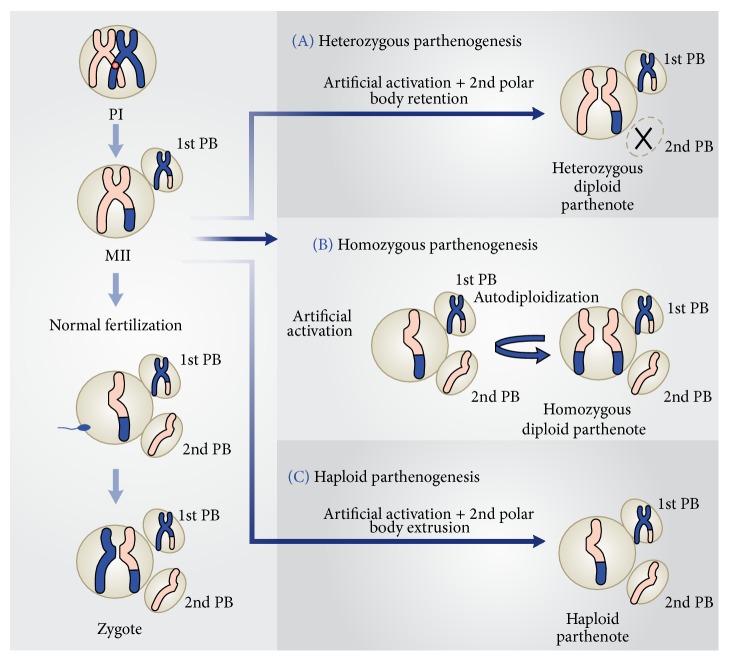
Meiosis and embryonic results after normal fertilization and different artificial activation protocols.

Arrested nonfertilized metaphase II (MII) oocytes will remain at this stage until a stimulus, which may come from the fertilizing spermatozoon or from an artificial agent (Figure 2), triggers intracytoplasmic Ca^2+^ rises and meiosis resumption. Intracellular Ca^2+^ oscillations will inhibit the action of the metaphase promoting factor (MPF) and the cytostatic factor (CSF) and lead to metaphase/anaphase transition, segregation of sister chromatids, and extrusion of second polar body. The importance of these Ca^2+^ transients for oocyte activation was shown by the prevention of the intracellular elevation of Ca^2+^ after sperm penetration by preloading the oocytes with the Ca^2+^ chelator BAPTA 1-AM [6]. In the absence of any intracellular Ca^2+^ increase, activation and subsequent embryo development failed to occur. In mammals, intracellular Ca^2+^ transients are triggered by a putative sperm factor, the testis specific phospholipase C-*zeta* (plc-*zeta*), or the newly described postacrosomal sheath WW domain-binding protein PAWP released by the sperm during the normal fertilization process [7]. Regardless of the exact nature of the sperm factor responsible for eliciting the Ca^2+^ oscillations, its presence is paramount for successful oocyte activation and embryonic development. The activating agents used for parthenogenetic activation may mimic the sperm bound stimuli to trigger Ca^2+^ release from the endoplasmic reticulum, exit from meiotic arrest, and start embryonic development.

### 2.2. Techniques of Parthenogenetic Activation

A wide range of artificial stimuli have been employed as agents to trigger the activation process in oocytes of several animal species. All the techniques which have been described for artificial activation can be divided into two major groups, depending on the nature of the activating stimulus, that is, chemical or physical agents. Except for Sr^2+^ [6], all other artificial agents that have been studied do not produce repetitive Ca^2+^ oscillations normally observed during fertilization. However, the single rise in intracellular Ca^2+^ that is produced by agents such as Ca^2+^ ionophore, ionomycin, or ethanol is adequate to trigger the meiotic resumption and cortical granule release. Specific oocyte activation protocols using strontium chloride (SrCl_2_) to induce continuous Ca^2+^ oscillations during exit from second meiosis and first embryonic mitosis seem to have a role in long-term embryonic events, such as the number of cells in the inner cell mass (ICM) and trophectoderm of the resulting blastocyst [8].

Despite the fact that various methods have been investigated for parthenogenetically activating oocytes through intracytoplasmic Ca^2+^ elevations, it seems that there is not a single method that activates oocytes from every species studied. Thus, parthenogenetic activation strategies have presented varying degrees of success, regarding activation rates and subsequent embryonic development according to the protocol employed and the species.

Figure 2 shows how embryos are generated after fertilization of an MII oocyte and the three possible parthenotes that may result according to the activation protocol employed to stimulate exit from MII, in the absence of a fertilizing spermatozoon.

In the first instance (Figure 2(A)), treatment with an activating agent such as SrCl_2_, ethanol, Ca^2+^ ionophore, or ionomycin is followed by another chemical, for instance, 6-DMAP (broad protein synthesis inhibitor) or cytochalasin B or D (inhibitors of actin filaments polymerization), which blocks second polar body (PB2) extrusion. Thus, the resulting parthenote is a “pseudodiploid” heterozygous embryo containing the two sister chromatids of each maternal chromosome present in the MII oocyte.

Alternative activation methodology may allow PB2 extrusion and the resulting parthenote containing a single copy of the sister chromatids undergoes spontaneous diploidization giving rise to a completely homozygous embryo (Figure 2(B)).

Last, the activating agent induces exit from MII and PB2 extrusion, without diploidization (Figure 2(C)). The result of this strategy is a haploid zygote, which develops into a haploid parthenote. However, in this instance, embryo development to the blastocyst stage is generally very low (Bos-Mikich, personal communication).

High oocyte activation and blastocyst formation rates were achieved, when mouse oocytes were exposed to Ca^2+^-free medium supplemented with 10 mM SrCl_2_ for 2 hours [6, 9]. However, similar success rates were not achieved when the same protocol was employed to activate human and bovine oocytes [10].

On the other hand, bovine oocytes presented high activation rates and development to the blastocyst stage, when exposed to the Ca^2+^ salt ionomycin for 5 minutes, followed by 2 mM 6-dimethylaminopurine (6-DMAP) for 3.5 hours [11]. This last activating method has been employed to generate parthenogenetic bovine pluripotent embryonic stem cell, resulting in high rates of derivation and proliferation of primary embryonic stem cell colonies [12].

### 2.3. Activating Protocols for Fresh Human Oocytes

Human oocytes have been artificially activated mainly with the aim to generate blastocysts for the creation of parthenogenetic human embryonic stem cell (phESC) lines [13]. Notably, parthenogenetic embryo development and ESC derivation efficiencies are similar to those of sperm fertilized counterparts [14].

The first phESC lines were created using Ca^2+^ ionomycin together with 6-DMAP [15]. In that study, fresh oocytes were obtained from shared oocyte donation cycles and the activation procedure took place few hours (3-4 hours) after collection. Twenty-three blastocysts (50%) were generated, from which six phESC lines were established. Mai et al. [14] described an activation methodology that combines electrical and chemical stimuli using ionomycin and 6-DMAP, for the artificial activation of fresh oocytes. Despite the fact that their parthenogenetic blastocyst rate was lower (21%) than the previous report [15], the inner cell mass of three blastocysts was used for the successful derivation of two new parthenogenetic stem cell lines. Blastocyst formation rates also vary considerably in ART cycles, depending on several factors such as the age of the patient, ovarian stimulation protocol, and culture conditions, among others. Thus, it is not surprising that reports on developmental rates of human parthenogenetic embryos vary between studies, as the age of the donors and the stimulation protocols may differ considerably, affecting the final blastocyst rates and consequently their ICM quality.

### 2.4. Activation of Failure to Fertilize Human Oocytes

The detection of nonfertilized oocytes after insemination is not uncommon in ART treatments, both due to gamete immaturity and unknown factor that led to a failure to fertilize (FF). Most frequently, FF occurs after IVF. In this situation, the oocyte will remain arrested in MII. Rescue insemination by ICSI, few hours after detection of FF, has been proposed as a valid alternative to induce the creation of zygotes from the nonfertilized oocytes [16–19]. There is, however, the underlying risk of chromosome abnormalities in the resulting embryo, mainly because of oocyte aging from the collection time to the moment of reinsemination by ICSI, which may occur 30 hours (or more) after oocyte pick-up. Also, the resulting clinical gestation rates vary considerably from center to center, which hampers rescue insemination to be used as a routine procedure in infertility treatments.

On the other hand, it has been shown that unwanted, FF human oocytes retain their developmental potential and may generate viable blastocysts by exposure to ionomycin followed by 6-DMAP and a protein synthesis inhibitor, cyclohexamide [20]. The embryos presented a similar gene expression profiling and developmental potential as normally fertilized ones making the unwanted artificially activated oocytes an alternative route for the generation of human embryonic stem cell lines.

A recent study [21] compared the efficacy of SrCl_2_ or Ca^2+^ ionophore on the activation rates of 3-day-old FF human oocytes and observed that the treatment with the ionophore was more efficient in activating aged unfertilized oocytes and embryo development to the blastocyst stage. However, these reports on the activation of FF oocytes do not reflect the real parthenogenetic embryonic development potential of the human oocyte as the studies mentioned earlier [11, 14, 15], because, in these last instances, there is a high probability that a paternal genome is present in the ooplasm, considering that IVF or ICSI was previously performed.

As an adjunct procedure in ART cycles, two intracellular Ca^2+^ releasing agents, SrCl_2_, and ionomycin have been used aiming at activating oocytes during fertilization. In a series of nine cases of couples who have had total fertilization failure or very low fertilization rates in previous cycles, Kyono et al. [22] exposed oocytes soon after insemination by ICSI to chemical stimulation using SrCl_2_. Fertilization rates increased from 21.7% in the previous cycles to 64.5% in the cycles using SrCl_2_ artificial activation. Six pregnancies were established, four of which went to term. Five healthy children were born and their 1-year followup did not show any physical or neurological abnormality. In another study, the same group of authors employed the Ca^2+^ ionophore to activate zygotes generated by ICSI using globozoospermic sperm [23]. The alternative use of Ca^2+^ ionophore to activate the oocytes during fertilization and induce embryo development and gestation in cases of previous FF of human oocytes was also reported with relatively good success rates by other groups [24–27].

The exposure to SrCl_2_ or Ca^2+^ ionophore may have induced the release and oscillations of intracellular Ca^2+^ needed for oocyte exit from the meiotic arrest and embryonic development [6, 9]. However, the putative epigenetic effects of this procedure demands caution and more research on the procedure. Also, the long-term followup of the children born by this methodology is necessary, before it can be considered as a routine methodology in human ART programs.

### 2.5. Oocyte Activation after* In Vitro* Maturation

In classical, stimulated ART cycles, immature germinal vesicle (GV) or metaphase I (MI) oocytes are commonly collected together with the mature MII. The immature oocytes may be submitted to* in vitro* maturation and ICSI to produce embryos for reproductive purposes. Resulting implantation and pregnancy rates are generally poor and there are controversies on whether to use these immature oocytes and embryos for reproductive purposes [28]. However, attempts to* in vitro* mature GV or MI oocytes obtained from stimulated ovaries may yield MII oocytes and potentially parthenotes, under suitable IVM conditions. Liu et al. [29] have shown that cryopreserved GV or MI oocytes collected from stimulated cycles yield good rates of high quality blastocysts, after insemination by ICSI and exposure to the activating agent ethanol. Thus, immature oocytes collected from stimulated ovaries should not be neglected as an additional source of gametes for parthenogenetic activation. Research should focus on protocols devised specifically to improve their cytoplasmic and nuclear maturation to generate blastocysts with high quality ICMs.

On the other hand, IVM protocols to promote maturation of oocytes collected from unstimulated ovaries exist and they are employed in several fertility centers around the world, as an alternative to classical, stimulated cycles [30–32]. The present results show that implantation and gestation rates after IVM are close to those obtained in classical stimulated cycles. Thus, surplus immature oocytes obtained from IVM cycles or from oophorectomy, in patients who undergo sex reassignment surgery [33], for instance, may represent an important source of gametes for artificial parthenogenetic activation and blastocyst formation after exposure to adequate IVM conditions.

## 3. Genetic and Epigenetic Outcomes in Parthenogenetic Stem Cells

The uniparental origin of parthenogenetic SCs (pESCs) confers its great advantage of producing immunocompatible pESCs [34], an adequate alternative source of pluripotent SCs presenting the main features of biparental stem cells, for example, typical morphology, expression of the pluripotency markers, self-renewal, and cell fate determination [35]. Moreover, some lineages have been reported as homozygous for human leukocyte antigens and therefore may be useful for regenerative therapies for a large number of patients [36].

Many aspects related to the biology of these cell lines, however, still need to be elucidated. Probably one of the major concerns of using pESCs cell for regenerative medicine is the inability of parthenote mammal embryos to support full-term development. It is known already that the accurate establishment of epigenetic regulation and maintenance of genomic imprints during embryogenesis are essential for normal embryonic/placental development [37]. The genomic imprinting phenomenon is conserved amongst eutherian mammals [38, 39] and is believed to have an important role in the allocation of maternal resources to fetal growth [40, 41].

In parthenotes, the lack of sperm alleles leads to the transcription of both alleles of maternally expressed imprinted genes and the absence of paternally expressed imprinted genes, possibly causing the overexpression of imprinted genes often associated developmental abnormalities [42, 43]. Indeed, the importance of the parental origin of genes was demonstrated when Kono and collaborators [4] successfully produced viable parthenogenetic offspring in mice by correcting the imprinted genes Igf2/H19 dosage. Genetic manipulation of oocytes allowed authors to demonstrate the possibility of parthenote survival.

Although parthenogenetic embryos retain aberrant imprints and consequently cannot develop to term, established parthenogenetic ESCs were reported morphologically indistinguishable from ES derived from fertilized embryos and may also present normal gene expression or even correction genomic imprinting in chimeras, when pESCs were used in tissue contribution [44]. Hence, the possibility of using cells or tissues derived from parthenotes for therapeutic applications is still important and desired.

## 4. Parthenogenetic Stem Cells for Regenerative Medicine

Whereas bone marrow transplantation is an example of stem cell therapy that is in clinical use worldwide, the therapeutic use of pluripotent stem cells, such as human embryonic stem cells (hESCs) or phESCs, is still in its infancy. First clinical trials in the US using hESCs involved specific conditions such as macular degeneration [45]. The wider application of hESCs is limited due to their genetic background, which will most likely be divergent from a potential patient. phESCs may overcome this limitation presented by hESCs in autologous histocompatible transplantations, as they should be isogenic with the gamete donor. One particularly interesting characteristic of phESCs is the fact that they show frequent homozygosity in the major histocompatibility locus, which may allow efficient immune matching [46, 47]. Furthermore, detailed genetic analysis on five rhesus monkey pESC lines showed that high levels of heterozygosity are maintained at loci, which were polymorphic in the oocyte donors [36] (see Figure 2(B)). While no clinical trial has been reported so far, exciting findings on the potential therapeutic use in of pESCs have been reported in mice and primate experimental models. Espejel et al. [48] demonstrated that pESC differentiated into hepatocytes and provided normal liver function in adult mice with lethal liver failure due to deficiency of fumarylacetoacetate hydrolase (Fah). Also in mice, it has already been shown that pESC derivates supported long-term hematopoiesis [49] and integrated electrically into recipient myocardium, becoming functionally undistinguishable from the recipient cardiomyocytes [34]. Finally, primate pESCs that differentiated into dopamine neurons presented long-term survival after transplantation into brain (allograft), without any teratoma formation [50].

Taken together, these observations indicate that pESC lines have the potential to be used in regenerative medicine, not only for autologous transplantation into oocyte donors, but also for creation of a bank of histocompatible cell lines. However, the possibility of an interplay of epigenetic reprogramming on a monoparental background, together with the lack of paternal imprinted genes expression, cast caution on hpESCs therapeutic application. Thus, additional research is needed to ensure the safety of using parthenogenetically derived ESC lines in regenerative medicine.

### 4.1. Generation of Gametes from Stem Cells

Despite all the advances in infertility treatments since the birth of Louse Brown in 1978, one challenging situation that remains to be overcome by ART is the patients with complete absence of gametes or gonads, who cannot have their genetically related child. Presently, gamete donation is the available alternative for these patients to conceive their children. Unfortunately, a worldwide shortage of altruistic gamete donors has exposed an increasing transaction of donor sperm and oocytes across borders [51]. This uncomfortable, sometimes ethically questionable, situation should be highly benefitted by advances on the generation of sperm and oocytes by manipulation of their progenitor cells or somatic cells [52].

Tesarik [53] proposed more than a decade ago the creation of haploid MII oocytes by introducing female somatic cell nuclei into enucleated MII oocytes. These gametes would undergo second polar body extrusion and haploidization* in vitro*, but fundamental limitations of chromosome segregation and imprinting relegated this methodology to the realm of the “fantastic” [54]. However, a recent report demonstrated that the use of a specific medium (4i) together with growth factors and Rho-kinase inhibitor for the culture of human embryonic stem cells and human induced pluripotent stem cells leads to the formation of embryo bodies, from which primordial germ-like cells were generated [55].

In another elegant study by Duggal et al. [56], the authors demonstrated that the synergism between inductive signals facilitates primordial germ cell, directed specification of embryonic stem cells followed by a premeiotic induction, in the presence of adequate IVM culture conditions.

These findings represent important steps towards the development of novel therapies in cases where patients are entirely devoid of viable gametes. Considering that* in vitro* differentiation of hpESCs has already provided completely different cell types [57, 58], the ability of these cells to generate primordial germ cells and mature oocytes may soon become a reality, which would represent an enormous gain in infertility treatments and related research areas.

## 5. Conclusion

It is clear from the information presented here that parthenogenetic activation of human oocytes rescued from infertility treatments results in embryos which are comparable to their biparental counterparts. The use of human parthenogenetic embryos as a source of cells for the generation of pluripotent stem cell lines is feasible and should be given more relevance in the present ESC research scenario. Considering the large number of oocytes discarded in infertility treatments [59], the* in vitro* differentiation of human parthenogenetically derived clinical grade stem cells shall produce a wide range of cell types, which will eventually have many clinical applications, including ART procedures.
